# Evaluation of the Farming Potential of *Echinacea Angustifolia* DC. Accessions Grown in Italy by Root-Marker Compound Content and Morphological Trait Analyses

**DOI:** 10.3390/plants9070873

**Published:** 2020-07-09

**Authors:** Nicola Aiello, Arianna Marengo, Fabrizio Scartezzini, Pietro Fusani, Barbara Sgorbini, Patrizia Rubiolo, Cecilia Cagliero

**Affiliations:** 1 Council for Agricultural Research and Economics, Research Centre for Forestry and Wood, 38123 Trento, Italy; nicola.aiello@crea.gov.it (N.A.); fabrizio.scartezzini@crea.gov.it (F.S.); pietro.fusani@crea.gov.it (P.F.); 2 Department of Drug Science and Technology, University of Turin, Via Pietro Giuria 9, 10125 Turin, Italy; arianna.marengo@unito.it (A.M.); barbara.sgorbini@unito.it (B.S.); cecilia.cagliero@unito.it (C.C.)

**Keywords:** marker-compound quantification, cynarin, dodeca-2E,4E,8Z,10E/Z-tetraenoic isobutylamide, echinacoside, morphological traits, root weight

## Abstract

The *Echinacea* genus includes a number of species that are commercially employed for the preparation of herbal products. *Echinacea angustifolia* DC. is one of these and is widely used, mainly for its immunomodulating properties, as it contains a wide range of compounds that belong to different chemical classes. In particular, echinacoside, cynarin and lipophylic alkylamides are the main specialized metabolites of the roots and can be considered to be marker compounds. In this work, 65 *E. angustifolia* accessions have been compared in a field trial in Italy, with the aim of investigating the variability/stability of the weight and chemical composition of their roots in order to identify the accessions that are most promising for future genetic-improvement programs. The morphological characteristics of the aerial parts have also been investigated. Seventeen samples were discarded due to germination or plantlet-development issues. Seven of the remaining accessions were identified as being different *Echinacea* species after a combined phytochemical and morphological evaluation. The morphological traits of the epigeal part, the root weight and the chemical composition data of the 41 confirmed *E. angustifolia* accessions were submitted to multivariate statistical analysis and a moderately homogenous sample distribution, with low selected-marker variability, was observed. Good echinacoside content was detected in almost all roots (>0.5%). However, two groups of accessions stood out because of their interesting features: One group possessed small roots, but had a high concentration of marker compounds, while another had highly developed roots and a good amount of marker compounds. These accessions can therefore be exploited for future selection work.

## 1. Introduction

*The Echinacea* genus (Asteraceae family) is indigenous to the Great Plains of North America and encompasses a small number of herbaceous grassland perennial taxa. Increasing interest in *Echinacea* as a commercial herbal product, including in herbal teas, capsules, tablets, powders, tinctures and beverages, has promoted its field cultivation, which has also been extended to Europe. *E. angustifolia* DC. (narrow-leaved coneflower), *E. pallida* (Nutt.) Nutt. (pale coneflower) and *E. purpurea* (L.) Moench (purple coneflower) are the main popular species in the dietary supplement markets. The taxonomy of the genus is still controversial. Although they were first considered to be varieties, McGregor described them, in 1968, as separate species on the basis of morphological criteria, cultivation and hybridization experiments, and cytological features [1,2,3,4]. McKeown et al., 1999 also reports that the majority of the species of *Echinacea* are diploid (n = 11), but *E. pallida* and some populations of *E. angustifolia* var. *strigosa* are tetraploid (n = 22) [5]. The roots from *E. pallida* and *E. angustifolia* are commercially used, while the aerial parts of *E. purpurea* are also employed. *E. angustifolia* is exploited for the production of herbal medicines and pharmaceutical, cosmetic and veterinary products due to the variety of healthy properties associated with its root extracts (e.g., immuno-modulating, anti-inflammatory, antiviral and antioxidant) [2,6,7]. The biological properties can be attributed to the different compounds that characterize *E. angustifolia* root extract, e.g., caffeic acid derivatives, polysaccharides, glycoproteins, and alkamides [7,8]. A standardized extract called Polinacea^®^, which is composed of ≥4.0% echinacoside—a high molecular weight polysaccharide of ca. 20,000 Da, (≥5.0%)—and alkamides (isobutylamides) (≤0.1%), has been used for a range of experimental studies [9,10]. The most abundant chemical markers of *E. angustifolia* are cynarin and echinacoside, for caffeic acid derivatives, and dodeca-2E,4E,8Z,10E-tetraenoic acid isobutylamide, for the alkamides. Echinacoside is the proposed marker compound for the standardization and quality evaluation of *E. angustifolia* roots [7,11,12]. The European Pharmacopoeia 10th ed. (2020) fixed the minimum content of this molecule at 0.5% [13].

To the best of the authors’ knowledge, there are no selected, highly producing secondary metabolite accessions of *E. angustifolia* available on the market, and *Echinacea* species are sometimes subjected to misidentification and confusion. Moreover, farming activity is not carried out intensively for this species, probably because the species shows some unfavorable characteristics, such as low germination rate, slow development and low root-weight yield, compared to *E. pallida* and *E. simulata*. A range of different in-vitro and in-vivo cultivation strategies have been investigated for this species [3,11,14,15,16,17]. However, it is important that the phytochemical variability of *E. angustifolia* plants is evaluated in order to potentially obtain accessions that produce the compounds of interest at high levels. Previous studies have been conducted on the chemical and morphological variation of *E. angustifolia*, on the basis of wild or cultivated accessions, the latitude of cultivation and the selected part of the plant [8,12,16,18,19,20]. As it was very important that the study was extended to a larger number of samples, the aim of this work is to analyze sixty-five accessions of *E. angustifolia*, collected from various sources such as a germplasm bank, botanical gardens, seed companies, and cultivated in the farm of the Research Centre for Forestry and Wood of the Council for Agricultural Research and Economics in Trento (Italy). The root weight, phytochemical composition and morphological aerial-part characteristics of the obtained plantlets were investigated to verify the authenticity of the *E. angustifolia* accessions and to assess sample variability/stability for a potential future selection of the accessions that are most promising for genetic-improvement programs.

## 2. Results

Sixty-five *E. angustifolia* accessions (Table 1) were collected and used for experimental field cultivation. Five of them were discarded due to the absence of seed germination (n° 4,53,56,61,62), the remaining plantlets were transplanted in June of the first year and monitored. In autumn of the same year, 35 accessions with developed stems, ranging from 1 to 33 in number, and 25 accessions without stems, with plants at the rosette stage, were registered. Twenty-six accessions had stems with open flower heads (from 1 to 15 flowered plants per accession) (Table S1). In June of the second year, 12 accessions (n° 10,11,14,15,23,29,30,31,32,35,36) showed a high number of missing plants and they were therefore not considered for the further evaluations.

### 2.1. Marker-Compound Quantification and Morphological Confirmation of the Accessions

**Figure 1:**
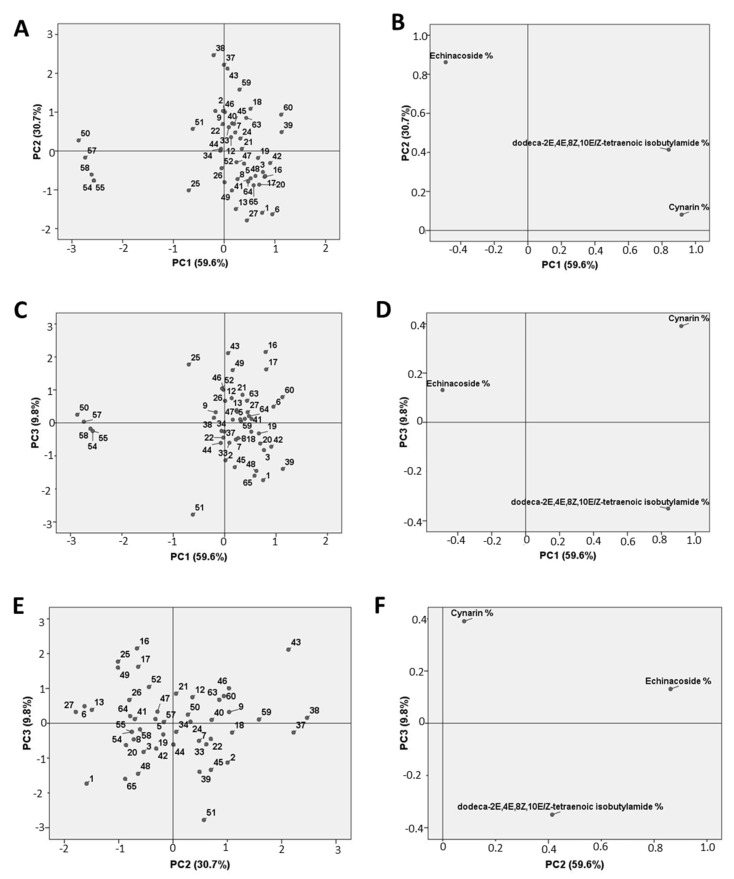
Principal Component Analysis (PCA) score plots and loading plots of 48 *E. angustifolia* accessions. Mean values of cynarin, echinacoside and dodeca-2E,4E,8Z,10E/Z-tetraenoic isobutylamide percentage content were used as variables. (**A**): score plot of PC1 vs. PC2; (**B**): loading plot of PC1 vs. PC2; (**C)**: score plot of PC1 vs. PC3; (**D)**: loading plot of PC1 vs. PC3; (**E)**: score plot of PC2 vs. PC3; (**F)**: loading plot of PC2 vs. PC3.

Three *E. angustifolia* marker compounds, namely cynarin, echinacoside and dodeca-2E,4E,8Z,10E/Z-tetraenoic isobutylamide, were identified and quantified in the roots of 3 or 4 individuals of each accession (Table S1). A representative chromatographic profile, obtained through the HPLC-PDA analysis, and the mass spectra of the commercial standards of the marker compounds are shown in Figure S1. The concentration of the three marker compounds ranged from 0 to 0.51% for cynarin, 0.39–2.86% for echinacoside and 0–0.9% for dodeca-2E,4E,8Z,10E/Z-tetraenoic isobutylamide (Table S2). The root weight was also recorded (from 1.1 to 53.3 g) (Table S2). Principal component analysis (PCA) was performed to better understand the variability/stability of these markers in the samples. The percentage contents of cynarin, echinacoside and dodeca-2E,4E,8Z,10E/Z-tetraenoic isobutylamide were considered as variables and, initially, all individuals were considered separately. In the score plot obtained for the first two components (85.8% of the total variation explained), the samples were homogenously dispersed with no positive or negative correlation with PC1 and PC2. A small cluster that negatively correlated with PC1 (accessions n° 50,54,55,57,58) was present (Figure S2). In order to simplify the data under investigation, the same statistical analysis was applied using the mean values for each accession (Figure 1). In this case, the analysis was expanded to the first three components. The combination of PC1 (59.6% of the variation explained) with both PC2 (30.7% of the variation explained) and PC3 (9.8% of the variation explained) gave results that were comparable to those reported in Figure S2, which had all the samples grouped together and a small separated cluster (accessions n° 50,54,55,57,58). The distribution of the variables suggests that there is negative correlation with the small cluster and cynarine and dodeca-2E,4E,8Z,10E/Z-tetraenoic isobutylamide content (Figure 1B,D).

**Figure 2:**
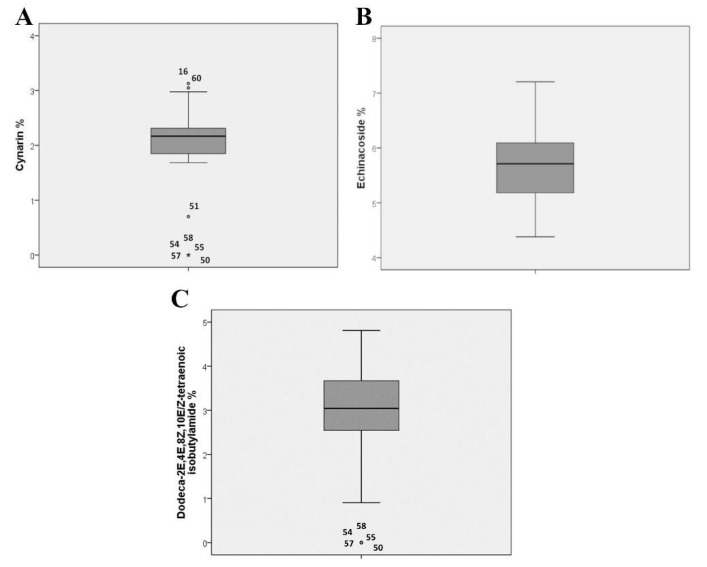
Box plots relative to cynarin (**A**), echinacoside, (**B**) and dodeca-2E,4E,8Z,10E/Z-tetraenoic isobutylamide; (**C**) mean content (%) in 48 *E. angustifolia* accessions. Values > 1.5 interquartile range (IQR) but < 3 IQR from the end of the box are labeled as outliers (dot), values >3 IQR from the end of a box are labeled as extreme (star).

The concentration of the marker compounds in each accession was thoroughly evaluated. Neither cynarin nor dodeca-2E,4E,8Z,10E/Z-tetraenoic isobutylamide were detected in the five accessions that were grouped together (Table S2). This difference is confirmed by the box plot analysis for cynarin and dodeca-2E,4E,8Z,10E/Z-tetraenoic isobutylamide (Figure 2). For cynarin, the box plot analysis highlights more outliers that are characterized by both lower (accession n° 51) and higher (accessions n° 16,60) quantities of this compound (Figure 2A).

**Figure 3:**
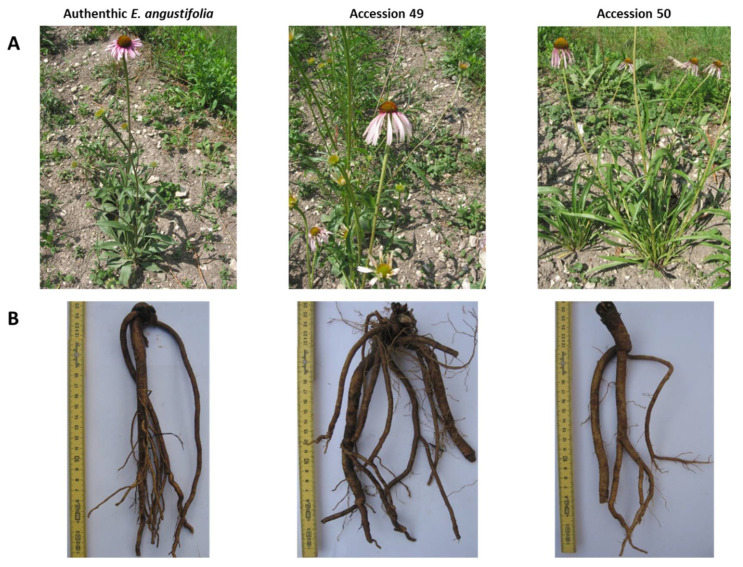
Morphological traits of an authentic *E. angustifolia* and of accessions 49 and 50. (**A**): aerial parts, (**B**): roots.

In order to better understand whether the phytochemical differences can be attributed to misleading species identification in the specific accessions, the main morphological traits of all the plants were considered. McGregor’s taxonomy reports nine species and four varieties for *Echinacea* genus [21]. Most of the considered accessions exhibited morphological characters that are typical of *E. angustifolia;* dark green leaves with a rough surface, green stem with short and dense hairs (a reddish green stem was observed for accessions n° 6,17,18,19,30,31,43 and long and dense hairs were recorded for accessions n° 1,2,3,7,8). The color of the ray flowers was deep pink in most of the accessions, followed by pink (1, 2, 3, 6, 7, 9, 13, and 16) and deep pink-fuchsia (46, 47, 48, 52, 59, 60, 63, 64, and 65). The teeth of the ray flowers were >2 mm in 27 accessions, between 1 and 2 mm in 12 accessions (3, 5, 7, 8, 13, 17, 19, 20, 24, 27, 33 and 34) and <1 mm in 2 accessions (9 and 16). At the same time, untypical morphological traits were recorded for accessions 49, 50, 51, 54, 55, 57, and 58. In particular, n° 49, 54, 55, 57, and 58 showed morphological characteristics that are typical of *E. simulata* or hybrids between that and *E. angustifolia* (tall plant, lanceolate leaves with long petiole, yellow pollen, long and dark pink-fuchsia ray flowers). The recorded characters of accession n° 51 were typical of *E. pallida* (tall plant, lanceolate leaves with long petiole, long and light pink ray flowers, whitish pollen). The morphological traits of n° 50 were typical of *E. paradoxa* var. *neglecta* (tall plants, leaves with a smooth and light green, pink ray flowers, yellow pollen) (Figure 3). The combination of the quantification results and this first morphological evaluation led to accessions n° 49,50,51,54,55,57 and 58 being excluded in order to evaluate the variability/stability of those considered to be authentic *E. angustifolia* accessions.

### 2.2. Phytochemical Evaluation and Morpho-Quantitative Characteristics of E. Angustifolia Accessions

The variability of cynarin, echinacoside and dodeca-2E,4E,8Z,10E/Z-tetraenoic isobutylamide concentrations in the remaining 41 *E. angustifolia* accessions was evaluated using PCA. Three principal components were selected and a homogenous sample distribution was observed, without the formation of distinctive clusters (Figure S3), as reported in the score plot. Some accessions that were further separated from the main central group were either characterized by higher amounts of cynarin (accessions n° 16,17,60), or lower percentages of dodeca-2E,4E,8Z,10E/Z-tetraenoic isobutylamide (accession n° 25), as confirmed by the box plots (Figure S4). A further investigation was performed by combining phytochemical data with agronomic features (i.e., number of germinated/developed plants) and a deeper morpho-quantitative analysis. The eight considered morphological traits varied in the accessions (Table S3). Accessions n° 6,5,18,25,17, and 45 were higher than accessions n° 2,8,21,27,34,37,38,39,40,43,44,46,47, and 48. The length and width of basal rosette leaves were variable: total length (petiole included) varied between 19.3 cm (accession 12) and 9.1 (accession 37), and width between 3.3 cm (accession 17) and 1.4 (accession 37). The shape of the leaf blade was also very variable as its “leaf length/width ratio” varied from 5.2 to 11.8. The leaf shape of accessions 25, 22, 9, and 24 was linear-lanceolate, while that of the remaining accessions was more or less lanceolate. The stems per plant varied from 1 (accession 64) to 12.7 (accession 13), while the number of flower heads per plant varied from 1.8 (accession 37) to 32.7 (accession 16). The main flower head diameter (capitulum and ray flowers) of accessions n° 44,45,52, and 42 was higher than that of accessions n° 9, 13, and 39. The number of ray flowers on the main flower head per plant varied from 14.3 (accession 16) to 22.2 (accession 63).

**Figure 4:**
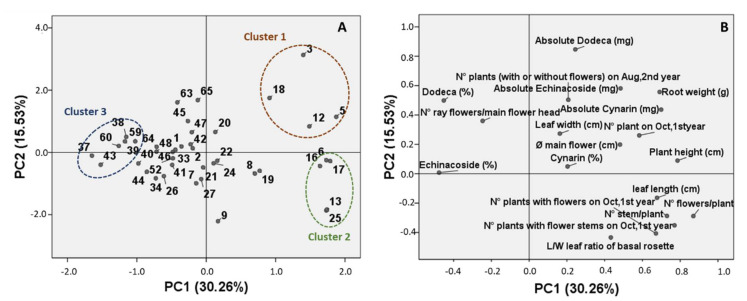
Principal Component Analysis (PCA) of 41 authentic *E. angustifolia* accessions based on phytochemical, agronomic and morpho-quantitative characteristics. (**A**): score plots of the different accessions; (**B**): loading plots of the variables.

Further elaborations (PCA and HCA, hierarchical cluster analysis) were therefore carried out, and the agronomic and morphological features of the plants were considered together with the phytochemical profile of the roots (in terms of concentration and absolute yield of the marker compounds). The PCA loadings plot (Figure 4B) of the first two components (45.8% of the total variation explained) showed a positive correlation between PC1 and plant height, leaf length, root weight, and absolute amount of the marker compounds in the roots. A positive correlation was also observed with the number of germinated and developed plants. On the other hand, a negative correlation between PC1 and the concentrations of echinacoside and dodeca-2E,4E,8Z,10E/Z-tetraenoic isobutylamide is noticeable. The Pearson correlation coefficient calculation (Table S4) confirmed these results. In particular, the root weight is statistically (*p* < 0.05) positively correlated with plant height, leaf length, N° of stems and flower/plants, and absolute amount of all three marker compounds and number of transplanted plants, while it is statistically negatively correlated to the concentration of echinacoside.

**Figure 5:**
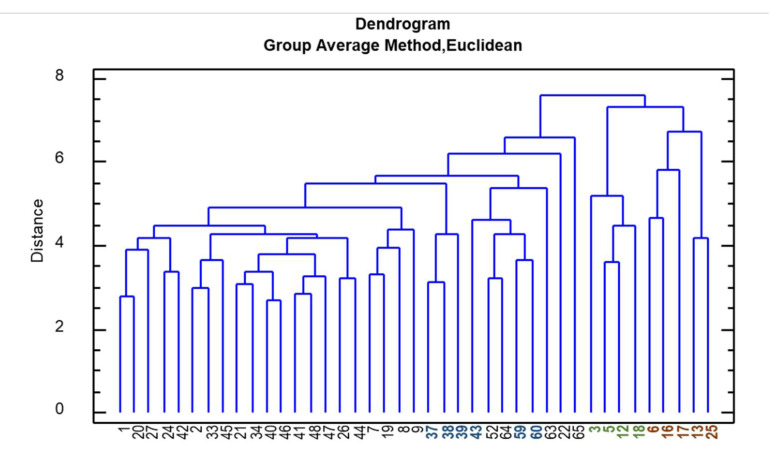
Hierarchical Cluster Analysis (HCA) of 41 authentic *E. angustifolia* accessions based on phytochemical, agronomic and morpho-quantitative characteristics.

The PCA scores plot (Figure 4A) showed a moderately homogenous sample distribution. However, three clusters can be identified: The first cluster includes accessions 3, 5, 12, and 18 and is characterized by tall plants with a high root weight and a high absolute amount of the marker compounds; the second cluster includes accessions 6, 13, 16, 17, and 25 and is characterized by tall plants, but with a low root weight and marker-compound content; finally, a third group of samples (accessions 37, 38, 39, 43, 59, and 60) is characterized by plants with a low plant height and root weight, but a high concentration of marker compounds. These groups are confirmed by the hierarchical cluster analysis reported in Figure 5.

## 3. Discussion

In this work, sixty-five *E. angustifolia* accessions were gathered from a germplasm bank, botanical gardens and seed companies and cultivated in a controlled experimental field. The first issue that was encountered was seed germination and the development of the plantlets. In fact, *E. angustifolia* is one of the most difficult echinacea species to grow successfully and is characterized by either poor or erratic germination, caused mainly by seed dormancy. Nevertheless, in line with previous works and thanks to treatment with gibberellic acid, 73.8% of accessions resulted in successful plantlet development [5,15,19,22]. The phytochemical profile of about 3/4 individuals from the resulting 48 accessions was evaluated first. Although few individuals were considered, the high number of the investigated accessions resulted in a high amount of data that were subjected to a multivariate data analysis (i.e. PCA, HCA) to obtain robust and evident results. Three marker compounds were selected. Echinacoside is the main caffeic acid derivative of *E. angustifolia* roots, but is also present in *E. pallida* roots. Cynarin, on the other hand, is typical of *E. angustifolia* roots. The roots of *E. angustifolia* contain large quantities of alkylamides, which are less abundant in *E. purpurea* aerial parts and mostly absent from the roots of *E. pallida*. Of the alkylamides, dodeca-2E,4E,8Z,10E/Z-tetraenoic acid isobutylamide is considered to be the main constituent [3,13,20,23]. The principal component analysis, based on the percentage content of the compounds as its variables, clearly identified five accessions (n° 50,54,55,57 and 58) as being classified as other *Echinacea* species. These results were confirmed by the morphological analysis. In addition, untypical morphological traits were observed for two other accessions (n° 49 and 51), which were not clearly chemically discriminated. *E. angustifolia* plants are 30–60 cm in height with one or more stems being mostly un-branched. The leaves are lanceolate to linear-lanceolate and the inflorescences are characterized by a cone-shaped disk and by purple, pale pink or barely white spreading ray flowers [24]. The misidentification and confusion of *Echinacea* species is common since they are morphologically similar and their systematics are still unclear [2,4,21,25]. The combination of the morphological and chemical characteristics of these accessions allowed us to assume that accessions n° 54,55,57, and 58, which are characterized, besides their morphological traits, by an absence of cynarin and dodeca-2E,4E,8Z,10E/Z-tetraenoic acid isobutylamide, can be identified as *E. simulata*. On the basis of its chemical profile, n° 49 is probably a hybrid and n° 50 and 51 can be identified as *E. paradoxa var neglecta* and *E. pallida*, respectively. [1,20,23]. For the accession 51, it would have been useful to make the analysis of the chromosome number, but being the only doubtful accession, this analysis was not carried out. We based the identification, as for the others accessions, on the morphological traits and the chemical profile that, in this case, revealed the absence of cynarin and presence of dodeca-2E,4E,8Z,10E/Z-tetraenoic acid isobutylamide in the roots. As reported by Jedrzejczyk (2020), the identification of *Echinacea* species is difficult, even from the biomolecular point of view; *E. angustifolia* is really very similar to other *Echinacea* species, such as *E. paradoxa* var. *neglecta* and *E. pallida*.

The European Pharmacopoeia (2020) indicates a minimum echinacoside content of 0.5%, and this parameter was confirmed by most of the 41 *E. angustifolia* analyzed roots. Only five individuals had a lower content (never below 0.39%). The content of cynarin and dodeca-2E,4E,8Z,10E/Z-tetraenoic acid isobutylamide was more variable. The obtained data and the variability of the abundance of these compounds were in agreement with previous works on 1-year-old roots from *E. angustifolia*; echinacoside was found in percentages ranging from 0.2 to 3.5%, 0.02–0.5% for cynarin, and 0.2–2.3% for dodeca-2E,4E,8Z,10E/Z-tetraenoic acid isobutylamide [8,12,16,18]. Similar results were obtained in samples harvested in different periods (e.g., 6 months, 3-year-old roots) [11,19,20,23,26,27]. Wu and coworkers (2009) have reported a higher dodeca-2E,4E,8Z,10E/Z-tetraenoic acid isobutylamide content (3.1%) in a 6-month-old root of *E. angustifolia*.

The multivariate statistical analysis of the marker-compound content in the considered authentic *E. angustifolia* accessions confirmed that the sample distribution was quite homogenous. However, when considering the root weight, the morphological traits of the aerial part and the root phytochemical features together, the correlations between some parameters can be noticed and some groups of accessions can be discriminated. In particular, the plants with well-developed aerial parts also present a good root–weight yield; these plants are also characterized by high vegetative success. On the other hand, the concentration of the marker compounds in these plants is lower. This can be attributed to the fact that the highest content of metabolites is found in the smaller and thinner roots, due to an accumulation of such constituents in the root cortex of the taproot [8].

These considerations and the results of the multivariate statistical elaborations allowed us to identify two groups of accessions as having interesting features. In particular, one group of accessions (i.e., accessions 37, 38, 39, 43, 59, and 60) is characterized by plants with small roots, but a considerably high concentration of marker compounds: For accessions 37 and 38, the concentration of echinacoside and dodeca-2E,4E,8Z,10E/Z-tetraenoic acid isobutylamide is higher than the mean values obtained for these compounds; for accession 39, the cynarin and dodeca-2E,4E,8Z,10E/Z-tetraenoic acid isobutylamide concentrations are higher than the mean; for accession 43, the echinacoside and cynarin concentrations are higher than the mean; while for accessions 59 and 60, the concentrations of all three of the marker compounds are higher than the mean. These accessions can therefore become a reference quality standard. On the other hand, a second group of accessions (i.e., accessions 3, 5, 12 and 18) is characterized by highly-developed roots, and a, nevertheless, good amount of the marker compounds, not only in terms of absolute amount, but also in terms of their concentration: For accession 3, the dodeca-2E,4E,8Z,10E/Z-tetraenoic acid isobutylamide concentration is higher than the mean value for this compound; for accession 5, the cynarin concentration is higher than the mean; for accession 12, the cynarin and echinacoside concentrations are higher than the mean; while for accession 18, the concentrations of all three of the marker compounds are higher than the mean. This group of accessions shows good vegetative development and promising marker-compound content, at the same time, and can therefore be exploited for future selection works.

## 4. Materials and Methods

### 4.1. Chemicals

HPLC-grade acetonitrile (LC-MS grade), methanol and formic acid were obtained from Sigma Aldrich (Bellefonte, PA, USA). De-ionized water (18.2 MΩ cm) was obtained from a Milli-Q purifification system (Millipore, Bedford, MA, USA). Cynarin (purity > 96%), echinacoside (purity > 95%) and dodeca-2E,4E,8Z,10E/Z-tetraenoic isobutylamide (purity > 98%) were from Phytolab (Vestenbergsgreuth, Germany).

### 4.2. Plant Material and Morpho-Quantitative Evaluation

Seeds belonging to 65 *E. angustifolia* accessions were obtained from different sources, as listed in Table 1. Seeds were treated with gibberellic acid (0.2 g L^−1^) and sowed, at the end of March of the first year, into plastic boxes containing a mixture of a substrate for horticulture (Manna Flor PT, Manna, Bolzano, Italy) and sand (about 2/3 and 1/3 in volume, respectively) and kept in a greenhouse at approximately 8 °C. Manna Flor PT composition was as follows: white peat (H2-H4) 25%, black peat (H6-H9) 75% with pH (CaCl_2_) 5.2–6: fertilizer (14N-16P-18K-2Mg) 1 kg m^−3^; electrical conductivity 300–370 µs cm^−1^; total salinity 0.9–1.1 g L^−1^. At the end of April, the seedlings were transplanted into 72-cell plastic trays (Amprica, Mantova, Italy) filled with the same mixture and kept there until June. The absence of germination was observed for accessions n° 4,53,56,61,62.

The obtained plantlets were cultivated in the experimental farm of the Research Centre for Forestry and Wood, Council for Agricultural Research and Economics, in Trento, Italy, at 371 m above sea level (46°02′53.29 ″N; 11°08′49.42 ″E) for 2 years. The climate is temperate sub-continental (annual average minimum and maximum temperatures: 7.3 and 16.8 °C, respectively; a.a. rainfall 1141.2 mm; a.a. rainy days 92.5; reference period 1979–2019).

For the 60 germinated accessions, approximately 40 plants were transplanted into the field in June of the first year (50 × 25 cm, 8 plants m^2^, four rows per accession). The soil of the experimental trial was composed of sand (30%), silt (60%), clay (10%) at pH 7.6, calcareous, with total CaCO_3_ (63%), but normal content of the active CaCO_3_ (1.1%), and a high content of organic matter (5.8%), total N (0.29%), P_2_O_5_ assimilable (142 mg kg^−1^), and exchangeable K_2_O (263 mg kg^−1^). Mechanical weed control was performed between the rows, while it was carried out manually in the rows. Plants were watered as needed without the use of fertilizer. The number of plants with developed stems (flowered or not flowered) and the leaf length and width of basal rosettes (the latter data not shown) were recorded in October of the first year. A first preliminary morphological analysis was performed on the leaf color, color and presence/length of hairs on the stem, color of ray flowers of the main flower head, and the length of “teeth” of ray flowers of the main flower head (<1, 1–2, >2 mm). In June of the second year, for each accession, 10 plants were selected, when available, at the full bloom phase (R5.3–R5.5) [26], and the following main morphological parameters were measured: plant height, leaf length, leaf width and L/W leaf ratio of basal rosette (on three different leaves), n° of stems per plant, n° of flower heads per plant, main flower head diameter (capitulum and ray flowers), and n° of ray flowers per main flower head.

The roots of three or four plants per accession were collected in October of the second year (see Table S1). The single roots were weighed, cut and dried (40 °C for 48 h), and subjected to solvent extraction for subsequent phytochemical analysis.

### 4.3. Extraction of Plant Material

One hundred milligrams of each dried and ground root were submitted to ultrasonic extraction with 10 mL of methanol/water (70:30, *v/v*), twice, for 10 min each. The resulting two extracts were combined and centrifuged at 4000 rpm for 10 min. The supernatant was brought to a volume of 30 mL and filtered through a 13 mm diameter, 0.22 μm pore diameter, and hydrophilic PTFE syringe filter, prior to HPLC-PDA-MS/MS analysis. The repeatability of extract composition was evaluated on 35 accessions with the RSD% between three extracts < 5%. The efficiency of the extraction process was determined by calculating the recovery of the three markers, which were 119%, 112% and 101% for cynarin, echinacoside and dodeca-2E,4E,8Z,10E/Z-tetraenoic isobutylamide, respectively.

### 4.4. HPLC-PDA-MS/MS Analysis and Quantification

#### 4.4.1. Qualitative Analysis

Each extract (5 μL) was analyzed using a Shimadzu Nexera × 2 system coupled with a photodiode array detector SPD-M20A in series to a Shimadzu LCMS-8040 triple quadrupole system equipped with an electrospray ionization (ESI) source (Shimadzu, Dusseldorf, Germany) for the identification of the three markers.

Samples were analyzed using an Ascentis Express C18 column (15 cm ×2.1 mm, 2.7 μm, Supelco, Bellefonte, NC, USA) with water/formic acid (999:1, *v*/*v*) and acetonitrile/formic acid (999:1, *v*/*v*) as the mobile phases A and B, respectively. The flow rate was 0.4 mL/min and the column temperature was maintained at 30 °C. The gradient program was as follows: 5–25% B in 10 min, 25–40% B in 5 min, 40–80% B in 15 min, 80–100% B in 3 min, and 100% B for 1 min. UV spectra were acquired over the 220–450 nm wavelength range.

Mass spectrometer operative conditions were: heat block temperature, 200 °C; desolvation line (DL) temperature, 230 °C; nebulizer gas (N_2_) flow rate, 3 L/min; and drying gas (N_2_) flow rate: 15 L/min. Mass spectra were acquired both in positive and in negative full-scan mode, over the range 50–1000 *m/z*, event time 0.5 s.

The three markers were identified in the extracts by comparing their retention times, UV and MS spectra with those of authentic standards.

#### 4.4.2. Quantitative Analysis

Each extract (5 μL) was analyzed in triplicate using a Shimadzu UFLC XR (Shimadzu, Dusseldorf, Germany) equipped with a photodiode array detector SPD-M20A with the same column, mobile phases, flow rate, and gradient program as in the qualitative analysis (see Section 4.4.1).

UV spectra were acquired in the 220–450 nm wavelength range, and the resulting chromatograms were integrated at 325 nm for the quantification of echinacoside and cynarin, and at 254 nm for dodeca-2E,4E,8Z,10E/Z-tetraenoic isobutylamide.

Quantitation was performed using an external standard calibration method, and the results expressed as % weight/weight. The calibration curve of cynarin was built by analyzing it at five concentrations in the range 1–50 mg/L. For echinacoside, five concentrations in the range 1–100 mg/L were used, and for dodeca-2E,4E,8Z,10E/Z-tetraenoic isobutylamide, six concentrations in the range 0.5–100 mg/L were used. The determination coefficient (R^2^) was higher than 0.998 in all cases. Analytical performance was measured in terms of repeatability and intermediate precision (RSD% never exceeding 3%).

All data were processed using Lab Solution software, version 5.92 (Shimadzu, Dusseldorf, Germany).

### 4.5. Statistical Analysis

Multivariate analysis (PCA, principal component analysis and HCA, hierarchical cluster analysis) as well as Pearson correlation was carried out on the raw data; the clustering was based on Euclidean distance with the group average method used as fusion criterion. All the computations were evaluated using SPSS 15.0 (IBM Corporation, Armonk, NY, USA) software.

## 5. Conclusions

The results obtained for several accessions that were considered as *E. angustifolia* confirm, from one side, the difficulties in the cultivation and the correct identification of this species. The combination of the main morphological traits and the quantification of cynarin and dodeca-2E,4E,8Z,10E/Z-tetraenoic acid isobutylamide marker compounds can help in the recognition of other possible *Echinacea* species, although the taxonomy of this genus is still unclear. The *E. angustifolia* samples that are considered authentic have a similar chemical profile with acceptable echinacoside content, in agreement with the European Pharmacopoeia (2020). However, when combining root weight, the morphological traits of the epigeal part and phytochemical data, some accessions stand out thanks to their interesting features. In particular, a set of accessions with small roots, but a high concentration of marker compounds, can be considered a quality reference standard, while another group with highly-developed roots and a good amount of the marker compounds is a good compromise between the search for the optimal agronomic and phytochemical features. These accessions could therefore be exploited as starting materials for future work selection.

## Figures and Tables

**Table 1:** List of *E. angustifolia* accessions. The country of origin and the PI accession/other origin are indicated.

Accession N°	State	PI Accession/Other Origin
1	Nebraska, USA	Ames 03075
2	USA	Ames 31323
3	Nebraska, USA	PI 312814
4	Oklahoma, USA	PI 421332
5	Kansas	PI 597601
6	Oklahoma	PI 631267
7	Minnesota	PI 649026
8	Maine	PI 664823
9	Nebraska	PI 421331
10	Nebraska	PI 421372
11	Oklahoma	PI 631261
12	Oklahoma	PI 631268
13	Oklahoma	PI 631269
14	Oklahoma	PI 631270
15	Oklahoma	PI 631271
16	Oklahoma	PI 631272
17	Oklahoma	PI 631273
18	Iowa	PI 631283
19	Iowa	PI 631284
20	Iowa	PI 631285
21	Iowa	PI 631286
22	Iowa	PI 631287
23	Iowa	PI 631288
24	Iowa	PI 631289
25	Kansas	PI 631317
26	Kansas	PI 631318
27	Kansas	PI 631319
28	Iowa	PI 633650
29	Iowa	PI 633651
30	North Dakota	PI 633652
31	North Dakota	PI 633653
32	North Dakota	PI 633654
33	North Dakota	PI 633655
34	North Dakota	PI 633656
35	North Dakota	PI 633657
36	Montana	PI 636393
37	North Dakota	PI 636394
38	North Dakota	PI 636395
39	North Dakota	PI 636396
40	Iowa	PI 649027
41	Iowa	PI 649028
42	North Dakota	PI 649029
43	North Dakota	PI 649030
44	North Dakota	PI 649031
45	Oklahoma	PI 649032
46	South Dakota	PI 649033
47	North Dakota	PI 664824
48	Wyoming	PI 664825
49	Wyoming	PI 631266
50	Oklahoma, USA	PI 631320
51	Goodwood, ON, Canada	Richters Herbs
52	Oregon, USA	Horizon Herbs
53	Arnhem, Netherlands	Open Lucht Museum
54	Ulm, Germany	Botanic Garden
55	Bonn, Germany	Botanic Garden
56	Bonn, Germany	Botanic Garden
57	Talence, France	Botanic Garden
58	Italy	Veneto Farm
59	Trento, Italy	CREA-FL
60	Trento, Italy	CREA-FL
61	Trento, Italy	CREA-FL
62	Trento, Italy	CREA-FL
63	Cultus Lake, BC, Canada	Strobl Farm
64	Cultus Lake, BC, Canada	Strobl Farm
65	Cultus Lake, BC, Canada	Strobl Farm

## Supplementary Materials

The following are available online at https://www.mdpi.com/2223-7747/9/7/873/s1. Figure S1: HPLC-PDA-MS/MS profiles of *E. angustifolia* root extract and the commercial standards. (A): PDA profile of the *E. angustifolia* root extract (λ = 254 nm) and echinacoside, dodeca-2E,4E,8Z,10E/Z-tetraenoic isobutylamide and cynarin standards. (B): ESI^+^ and ESI^−^ mass spectra of echinacoside commercial standard acquired in scan mode, (C): ESI^+^ and ESI^−^ mass spectra of dodeca-2E,4E,8Z,10E/Z-tetraenoic isobutylamide commercial standard acquired in scan mode, (D): ESI^+^ and ESI^−^ mass spectra of cynarin commercial standard acquired in scan mode. Figure S2: Principal Component Analysis (PCA) score plot of individuals belonging to 48 *E. angustifolia* accessions, based on the cynarin, echinacoside and dodeca-2E,4E,8Z,10E/Z-tetraenoic isobutylamide percentage content as variables.: Figure S3: Principal Component Analysis (PCA) score plot of 41 authentic *E. angustifolia* accessions. Mean values of cynarin, echinacoside and dodeca-2E,4E,8Z,10E/Z-tetraenoic isobutylamide percentage content were used as variables. Figure S4: Box plots relative to cynarin, echinacoside and dodeca-2E,4E,8Z,10E/Z-tetraenoic isobutylamide mean content (%) in 41 authentic *E. angustifolia* accessions. Table S1: Agronomic features for the investigated *E. angustifolia* accessions. Table S2: Cynarin (Cyn), echinacoside (Ech) and dodeca-2E,4E,8Z,10E/Z-tetraenoic isobutylamide (Dod) content (% on dry weight) in the root and root weight (RW) of different *E. angustifolia* accessions. Data are expressed as mean values and standard deviations (SD). Table S3: Morpho-quantitative characteristics of different *E. angustifolia* accessions (H: height (cm); LL: Leaf length (cm); LW: Leaf width (cm); L/W: L/W leaf ratio of basal rosette; N°S: N° stems/plant; N°F: N° flowers/plant; Ø F: diameter of the main flower (cm); N°RF: N° ray flowers/main flower head). Data are expressed as mean values and standard deviations (SD). Table S4: Pearson’s correlation matrix between cynarin, echinacoside and Dodeca-2E,4E,8Z,10E/Z-tetraenoic isobutylamide % quantitation results (%) and morpho-quantitative variables.

## References

[1] Brown P.N., Chan M., Betz J.M. (2010). Optimization and single-laboratory validation study of a high-performance liquid chromatography (HPLC) method for the determination of phenolic Echinacea constituents. Anal. Bioanal. Chem..

[2] Mistríková I., Vaverková Š. (2007). Morphology and anatomy of Echinacea purpurea, E. angustifolia, E. pallida and Parthenium integrifolium. Biologia.

[3] Raclariu A.C., Ţebrencu C.E., Ichim M.C., Ciupercǎ O.T., Brysting A.K., de Boer H. (2018). What’s in the box? Authentication of Echinacea herbal products using DNA metabarcoding and HPTLC. Phytomedicine.

[4] Binns S.E., Baum B.R., Arnason J.T. (2002). A taxonomic revision of echinacea (Asteraceae: Heliantheae). Syst. Bot..

[5] Mckeown K.A. (1999). A Review of the Taxonomy of the Genus Echinacea.

[6] Dall’Acqua S., Grabnar I., Verardo R., Klaric E., Marchionni L., Luidy-Imada E., Sut S., Agostinis C., Bulla R., Perissutti B. (2019). Combined extracts of Echinacea angustifolia DC. and Zingiber officinale Roscoe in softgel capsules: Pharmacokinetics and immunomodulatory effects assessed by gene expression profiling. Phytomedicine.

[7] Barnes J., Anderson L.A., Gibbons S., Phillipson J.D. (2005). Echinacea species ( Echinacea angustifolia (DC.) Hell., Echinacea pallida (Nutt.) Nutt., Echinacea purpurea (L.) Moench): A review of their chemistry, pharmacology and clinical properties. J. Pharm. Pharmacol..

[8] Aiello N., Carlini A., Scartezzini F., Fusani P., Berto C., Dall’Acqua S. (2015). Harvest in different years of growth influences chemical composition of Echinacea angustifolia roots. Ind. Crops Prod..

[9] Dapas B., Dall’Acqua S., Bulla R., Agostinis C., Perissutti B., Invernizzi S., Grassi G., Voinovich D. (2014). Immunomodulation mediated by a herbal syrup containing a standardized Echinacea root extract: A pilot study in healthy human subjects on cytokine gene expression. Phytomedicine.

[10] Starvaggi Cucuzza L., Motta M., Accornero P., Baratta M. (2008). Effect of Echinacea augustifolia extract on cell viability and differentiation in mammary epithelial cells. Phytomedicine.

[11] Pellati F., Benvenuti S., Melegari M., Lasseigne T. (2005). Variability in the composition of anti-oxidant compounds in Echinacea species by HPLC. Phytochem. Anal..

[12] Willick I.R., Barl B., Tanino K.K. (2020). Effect of latitude on narrow-leaved purple coneflower ( Echinacea angustifolia DC.) yield and phytochemical quality. Can. J. Plant. Sci..

[13] European Pharmacopoeia (2020). Narrow-Leaved Coneflower Echinaceae Angustifoliae Radix.

[14] Lucchesini M., Pacifici S., Maggini R., Pardossi A., Mensuali Sodi A. (2019). A novel microfloating culture system for the in vitro rooting of Echinacea angustifolia D.C.: Photosynthetic performance and production of caffeic acid derivatives. Plant. Cell. Tissue Organ. Cult..

[15] Lucchesini M., Bertoli A., Mensuali-Sodi A., Pistelli L. (2009). Establishment of in vitro tissue cultures from Echinacea angustifolia D.C. adult plants for the production of phytochemical compounds. Sci. Hortic..

[16] Aiello N., Carlini A., Scartezzini F., Fusani P., Berto C., Dall’Acqua S. (2018). Effect of growth substrates on morpho-quantitative and qualitative characteristics of Echinacea angustifolia var. angustifolia roots. J. Herbs Spices Med. Plants.

[17] Galambosi B. (2004). Cultivation in Europe. The Genus Echinacea.

[18] Dall’Acqua S., Nicola A., Fabrizio S., Valentina A., Gabbriella I. (2010). Analysis of highly secondary-metabolite producing roots and flowers of two Echinacea angustifolia DC. var. angustifolia accessions. Ind. Crops Prod..

[19] Binns S.E., Arnason J.T., Baum B.R. (2002). Phytochemical variation within populations of Echinacea angustifolia (Asteraceae). Biochem. Syst. Ecol..

[20] Wu L., Dixon P., Nikolau B., Kraus G., Widrlechner M., Wurtele E. (2009). Metabolic profiling of Echinacea genotypes and a test of alternative taxonomic treatments. Planta Med..

[21] McGregor R.L. (1968). The taxonomy of the genus Echinacea (Compositae). Univ. Kansas Sci. Bull..

[22] Chuanren D., Bochu W., Wanqian L., Jing C., Jie L., Huan Z. (2004). Effect of chemical and physical factors to improve the germination rate of Echinacea angustifolia seeds. Coll. Surf. B Biointerf..

[23] Pietta P., Mauri P., Fuzzati N. (2004). Analytical Profiles of Echinacea Species. The Genus Echinacea.

[24] Greenfield J., Davis J. Echinacea (Echinacea Angustifolia) 2012. https://newcropsorganics.ces.ncsu.edu/wp-content/uploads/2017/06/Echinacea-Angustifolia.pdf.

[25] Jedrzejczyk I. (2020). Genome size and SCoT markers as tools for identification and genetic diversity assessment in Echinacea genus. Ind. Crops Prod..

[26] Berti M., Wilckens R., Fischer S., Hevia F. (2002). Effect of harvest season, nitrogen, phosphorus and potassium on root yield, echinacoside and alkylamides in Echinacea angustifolia L. in Chile. Acta Hortic..

[27] Kabganian R., Carrier D.J., Sokansanj S. (2008). Drying of Echinacea angustifolia roots. J. Herbs Spices Med. Plants.

