# Supplementary material for: Evaluation of the Farming Potential of Echinacea Angustifolia DC. Accessions Grown in Italy by Root-Marker Compound Content and Morphological Trait Analyses
Source: Plants (Basel). 2020 Jul 9;9(7):873. doi: 10.3390/plants9070873 (PMC7412158; doi:10.3390/plants9070873)
Supplement: Supplementary file 1 [file plants-09-00873-s001.pdf]

SUPPLEMENTARY MATERIAL

Evaluation of the farming potential of *Echinacea angustifolia* DC. accessions grown in Italy by root-marker compound content and morphological trait analyses

Nicola Aiello [1a], Arianna Marengo [2a], Fabrizio Scartezzini [1], Pietro Fusani [1], Barbara Sgorbini [2], Patrizia Rubiolo [2b*] and Cecilia Cagliero [2b]

[1] Council for Agricultural Research and Economics, Research Centre for Forestry and Wood, Trento, Italy nicola.aiello@crea.gov.it ; nicola.aiello@crea.gov.it (N.A.), fabrizio.scartezzini@crea.gov.it (F.S.), pietro.fusani@crea.gov.it (P.F.)

[2] Department of Drug Science and Technology, University of Torino, Via Pietro Giuria 9,10125 Torino, Italy; arianna.marengo@unito.it (A.M.), barbara.sgorbini@unito.it (B.S.), cecilia.cagliero@unito.it (C.C.), patrizia.rubiolo@unito.it (P.R.)

* Correspondence: patrizia.rubiolo@unito.it; Tel.: +390116707173

[a] Both authors contributed equally to this work

[b] Both authors contributed equally to this work

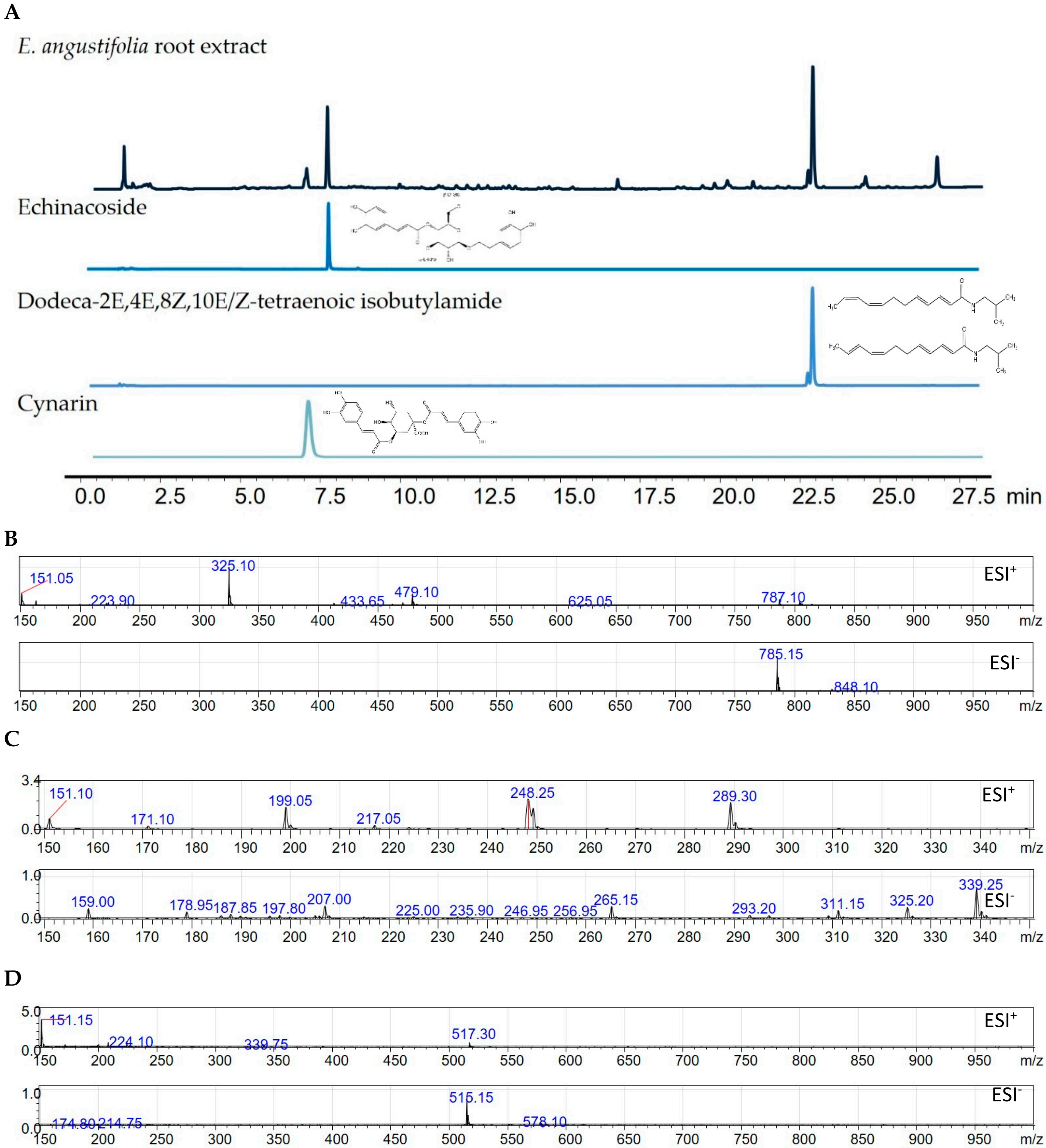

FigureS1: HPLC-PDA-MS/MS profiles of *E. angustifolia* root extract and the commercial standards. A: PDA profile of the *E. angustifolia* root extract (λ=254nm) and echinacoside, dodeca-2E,4E,8Z,10E/Z-tetraenoic isobutylamide and cynarin standards. B: ESI^+ and ESI^- mass spectra of echinacoside commercial standard acquired in scan mode, C: ESI^+ and ESI^- mass spectra of dodeca-2E,4E,8Z,10E/Z-tetraenoic isobutylamide commercial standard acquired in scan mode, D: ESI^+ and ESI^- mass spectra of cynarin commercial standard acquired in scan mode.

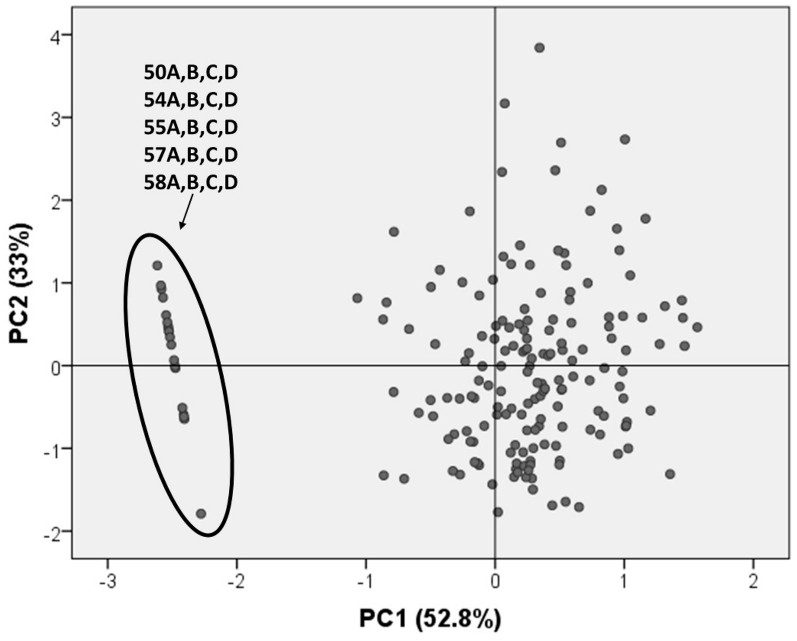

Figure S2: Principal Component Analysis (PCA) score plot of individuals belonging to 48 *E. angustifolia* accessions based on the cynarin, echinacoside and dodeca-2E,4E,8Z,10E/Z-tetraenoic isobutylamide percentage content as variables.

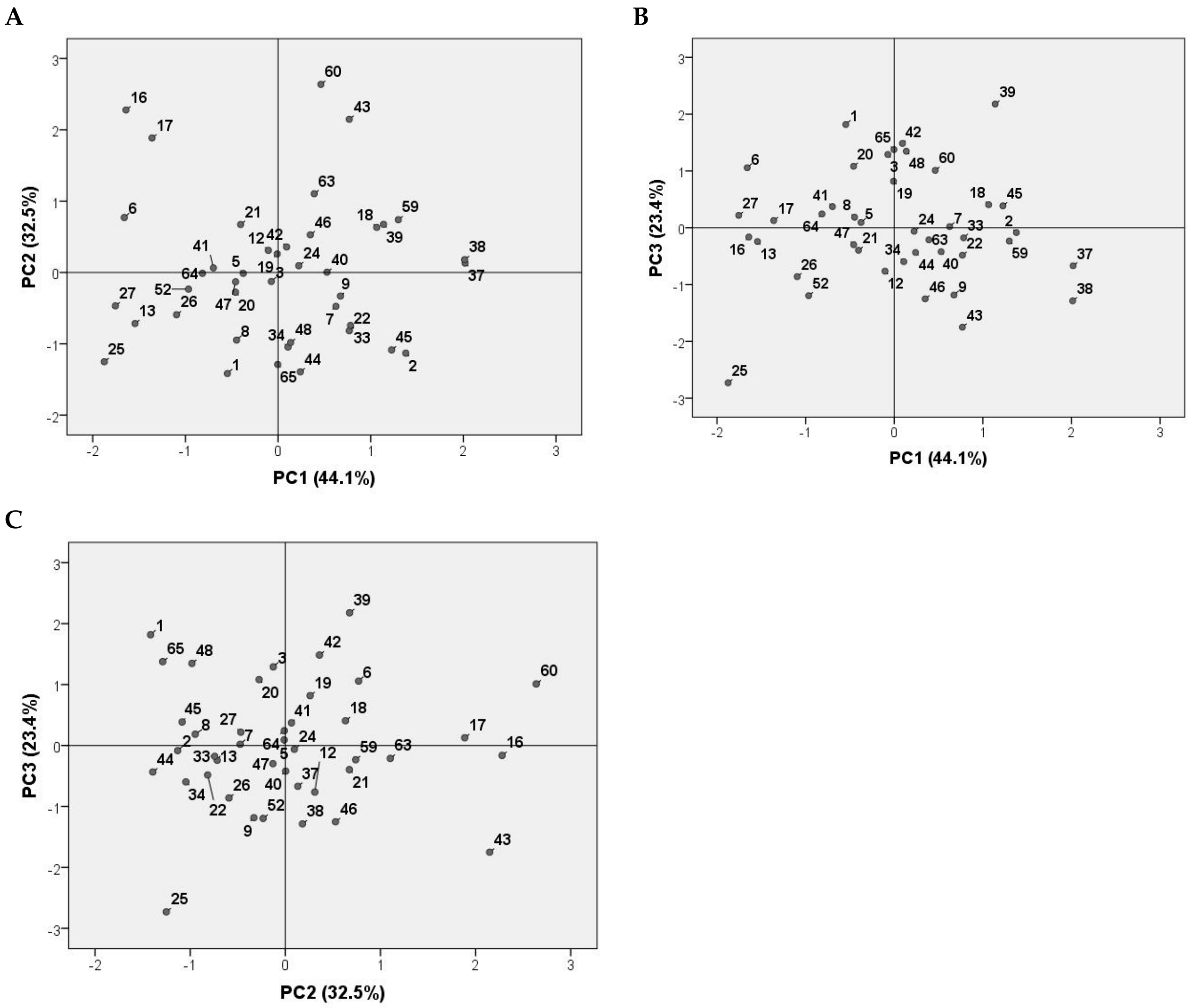

Figure S3: Principal Component Analysis (PCA) score plot (A: PC1 *vs*. PC2, B: PC1 *vs*. PC3, C: PC2 *vs*. PC3) of 41 authentic *E. angustifolia* accessions. Mean values of cynarin, echinacoside and dodeca-2E,4E,8Z,10E/Z-tetraenoic isobutylamide percentage content were used as variables.

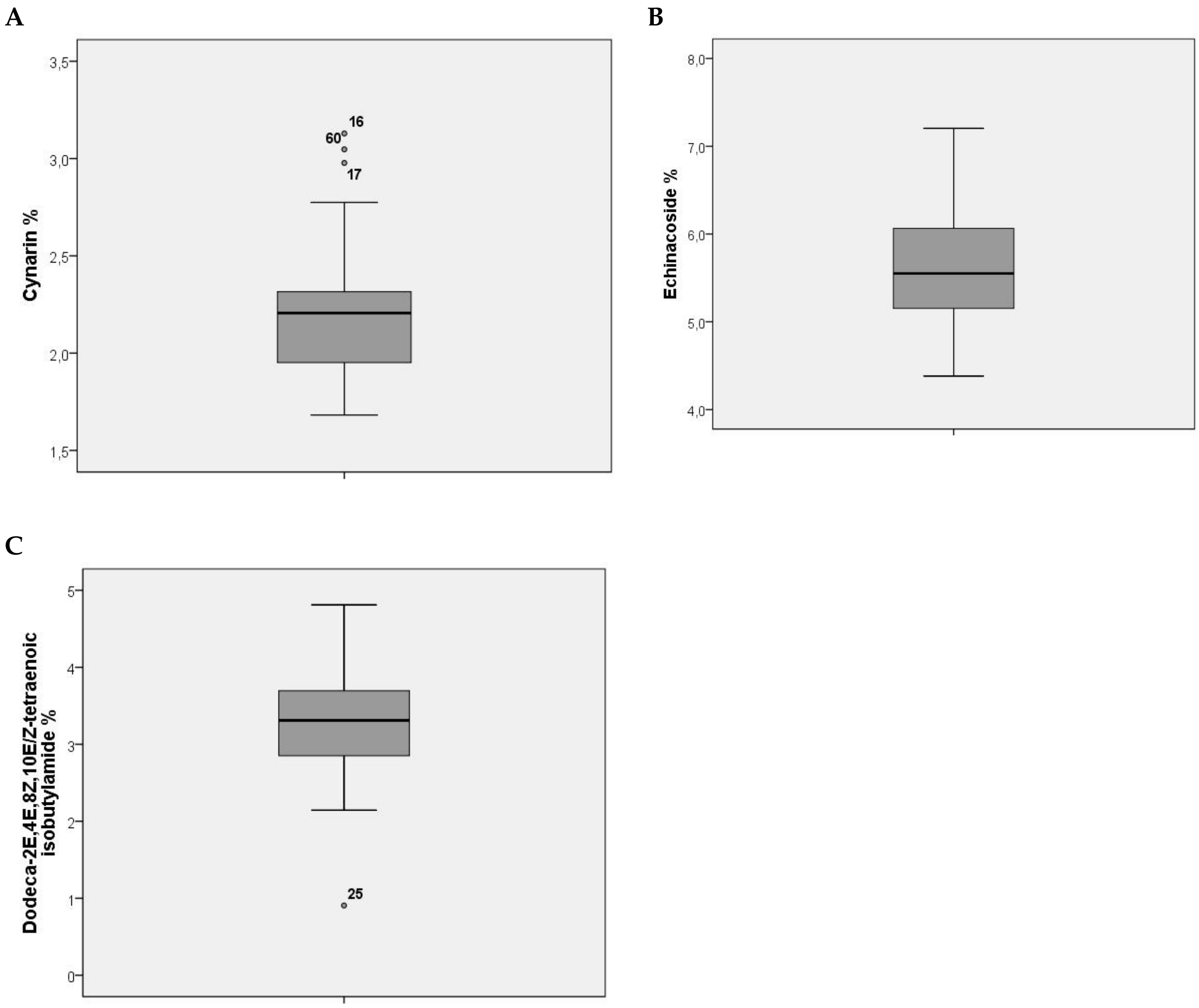

Figure S4: Box plots relative to cynarin (A), echinacoside (B) and dodeca-2E,4E,8Z,10E/Z-tetraenoic isobutylamide (C) mean content (%) in 41 authentic *E. angustifolia* accessions.

Table S1: Agronomic features for the investigated *E. angustifolia* accessions

Accession (n°)	Plants transplantated (June, first year) (n°)	Plants present (October, first year) (n°)	Plants with stems (October, first year) (n°)	Plants with flowers (October, first year) (n°)	Plants assessed (June, second year) (n°)	N° plants on August, second year (with or without flowers)	Roots analyzed (n°)
1	32	14	2	2	10	12	4
2	40	20	6	5	10	15	4
3	40	32	0	0	10	22	4
4							0 seedlings obtained
5	40	23	15	4	10	12	4
6	40	30	18	4	9	10	4
7	28	14	7	3	6	7	4
8	40	25	12	7	10	6	4
9	24	12	10	5	3	3	3
10	12	6	3	3	2		0 (1 root remained)
11	40	32	31	15	5		0 (2 roots remained)
12	40	23	9	0	7	10	4
13	40	38	23	5	10	14	4
14	34	17	15	7	3		0 (1 root remained)
15	40	19	16	6	2		0 (1 root remained)
16	40	23	16	4	3	6	4
17	40	38	33	10	4	8	4
18	40	24	10	3	10	19	4
19	40	27	13	3	10	16	4
20	40	19	4	1	9	14	4
21	40	29	2	1	10	17	4
22	24	11	2	0	4	3	3
23	8	8	4	1	2		0 (2 roots remained)
24	27	12	3	0	6	6	3
25	40	28	16	8	10	18	4
26	18	10	2	0	9	7	4
27	20	10	3	1	7	6	3
28	24	10	0	0	4		0 (1 root remained)
29	9	4	2	1	2		0 (2 roots remained)
30	16	8	3	1	2		0 (1 root remained)
31	40	18	8	3	7		0 (2 roots remained)
32	33	16	6	4	9		0 (2 roots remained)
33	40	22	2	0	10	10	4
34	40	23	2	2	10	8	4
35	40	19	0	0	9		0 (2 roots remained)

36	34	14	0	0	5		0 (1 root remained)
37	35	16	0	0	9	11	3
38	40	19	0	0	7	7	3
39	40	20	1	0	9	10	4
40	21	11	0	0	6	7	4
41	40	20	0	0	9	11	4
42	19	9	0	0	7	8	4
43	8	5	3	0	3	4	4
44	26	11	11	0	10	9	4
45	40	40	1	0	10	17	4
46	40	13	0	0	10	13	4
47	40	27	0	0	10	23	4
48	40	21	0	0	9	13	4
49	43	23	0	0	10		4
50	40	36	0	0	10		4
51	10	10	0	0			4
52	10	10	0	0	9	8	3
53							0 seedlings obtained
54	40	40	0	0			4
55	10	7	0	0			4
56							0 seedlings obtained
57	21	19	0	0			4
58	40	38	0	0			4
59	12	11	0	0	3	9	4
60	8	8	0	0	7	8	4
61							0 seedlings obtained
62							0 seedlings obtained
63	34	29	0	0	6	10	4
64	14	12	0	0	3	9	4
65	40	37	0	0	10	34	4

Table S2: Cynarin (Cyn), echinacoside (Ech) and dodeca-2E,4E,8Z,10E/Z-tetraenoic isobutylamide (Dod) content (% on dry weight) in the roots and root weight (RW) of different *E. angustifolia* accessions. Data are expressed as mean values and standard deviations (SD).

Accession (replicates)	Cyn %	SD	Ech %	SD	Dod (%)	SD	RW	SD
1 (4)	0.10	0.03	0.58	0.14	0.38	0.06	7.2	2.9
2 (4)	0.09	0.02	1.16	0.60	0.45	0.10	5.6	2.1
3 (4)	0.15	0.05	0.78	0.47	0.42	0.19	16.7	9.1
5 (4)	0.15	0.07	0.88	0.07	0.28	0.09	14.0	4.1
6 (4)	0.22	0.10	0.61	0.13	0.24	0.08	9.0	7.9
7 (4)	0.12	0.01	1.04	0.42	0.38	0.13	3.8	1.1
8 (4)	0.12	0.04	0.81	0.33	0.27	0.06	8.3	5.5
9 (3)	0.12	0.07	1.23	0.24	0.28	0.26	3.7	2.1
12 (4)	0.16	0.03	1.07	0.52	0.25	0.04	12.6	6.8
13 (4)	0.14	0.03	0.70	0.12	0.15	0.06	5.6	2.5
16 (4)	0.30	0.18	0.83	0.54	0.18	0.04	8.2	6.9
17 (4)	0.27	0.06	0.82	0.22	0.22	0.13	7.9	5.8
18 (4)	0.16	0.05	1.14	0.18	0.49	0.19	9.4	3.5
19 (4)	0.16	0.04	0.87	0.24	0.39	0.03	5.3	3.3
20 (4)	0.15	0.08	0.73	0.24	0.35	0.12	8.9	0.8
21 (4)	0.18	0.06	0.98	0.69	0.25	0.08	4.3	2.4
22 (3)	0.10	0.04	1.12	0.25	0.34	0.36	8.7	3.5
24 (3)	0.15	0.01	1.02	0.23	0.33	0.10	7.4	3.9
25 (4)	0.11	0.03	0.92	0.54	0.02	0.01	8.2	5.8
26 (4)	0.14	0.05	0.85	0.22	0.15	0.07	5.2	1.5
27 (3)	0.15	0.04	0.63	0.08	0.16	0.07	8.5	3.2
33 (4)	0.11	0.02	1.08	0.09	0.37	0.06	5.1	2.5
34 (4)	0.10	0.05	1.00	0.44	0.26	0.09	3.9	2.7
37 (3)	0.12	0.03	1.47	0.94	0.50	0.17	3.6	2.3
38 (3)	0.12	0.15	1.57	1.12	0.44	0.20	5.1	1.9
39 (4)	0.17	0.06	0.91	0.19	0.70	0.17	4.1	3.3
40 (4)	0.14	0.07	1.12	0.35	0.34	0.24	5.5	3.1
41 (4)	0.16	0.03	0.80	0.22	0.27	0.18	4.2	1.7
42 (4)	0.17	0.04	0.81	0.26	0.47	0.27	6.5	1.8
43 (4)	0.23	0.10	1.52	0.35	0.29	0.04	3.3	2.3
44 (4)	0.09	0.02	0.98	0.39	0.28	0.08	4.2	2.1
45 (4)	0.09	0.04	1.06	0.41	0.48	0.23	5.6	2.5
46 (4)	0.16	0.07	1.24	0.36	0.26	0.18	6.1	1.8
47 (4)	0.15	0.07	0.91	0.44	0.24	0.09	7.3	3.8
48 (4)	0.11	0.05	0.76	0.19	0.43	0.11	4.8	2.0
49 (4)	0.19	0.02	0.82	0.26	0.10	0.03	10.6	3.6
50 (4)	0.00	0.00	1.41	0.35	0.00	0.00	12.2	2.6

51 (4)	0.02	0.01	1.09	0.36	0.41	0.05	13.8	8.6
52 (3)	0.15	0.07	0.94	0.32	0.14	0.07	6.6	4.1
54 (4)	0.00	0.00	1.12	0.41	0.00	0.00	40.4	11.6
55 (4)	0.00	0.00	1.12	0.16	0.00	0.00	19.9	5.7
57 (4)	0.00	0.00	1.28	0.29	0.00	0.00	13.7	6.2
58 (4)	0.00	0.00	1.16	0.20	0.00	0.00	25.9	15.0
59 (4)	0.16	0.03	1.29	0.60	0.46	0.16	5.5	1.8
60 (4)	0.28	0.11	1.07	0.06	0.52	0.08	3.5	2.8
63 (4)	0.19	0.11	1.13	0.71	0.36	0.12	8.1	2.6
64 (4)	0.16	0.05	0.79	0.25	0.25	0.13	6.7	2.2
65 (4)	0.10	0.06	0.72	0.21	0.41	0.10	7.0	2.4
Average	0.13	0.05	1.00	0.34	0.29	0.11	8.6	3.9

Table S3: Morpho-quantitative characteristics of different *E. angustifolia* accessions (H:height (cm); LL: Leaf length (cm); LW: Leaf width (cm); L/W: L/W leaf ratio of basal rosette; N°S: N° stems/plant; N°F: N° flowers/plant; Ø F: diameter of the main flower (cm); N°RF: N° ray flowers/main flower head). Data are expressed as mean values and standard deviations (SD).

**Accession (replicates)**	**H**	**SD**	**LL**	**SD**	**LW**	**SD**	**L/W**	**SD**	**N° S**	**SD**	**N° F**	**SD**	**Ø F**	**SD**	**N° RF**	**SD**
1 (10)	47.5	9.8	11.2	3.4	2.2	0.6	5.5	2.2	7.3	4.2	10.5	7.4	8.9	1.3	17.5	4.6
2 (10)	47.2	5.8	13.2	3.8	2.2	0.8	6.7	2.7	8.3	4.3	10.7	9.4	9.1	1.6	19.5	4.5
3 (10)	53.9	5.2	15.2	2.8	2.1	0.4	7.3	1.9	9.8	4.8	12.6	6.4	8.6	2.0	17.8	3.2
5 (10)	69.2	8.8	15.8	2.7	2.4	1.0	7.3	2.3	9.9	8.0	24.0	17.9	8.3	1.8	18.2	3.9
6 (9)	77.9	19.1	15.3	5.1	2.1	0.9	8.2	2.8	9.7	7.3	26.0	14.2	8.8	2.0	18.7	5.2
7 (6)	57.6	11.9	16.5	2.9	2.2	0.6	7.7	1.4	6.7	5.0	14.2	11.1	9.0	2.7	17.5	3.2
8 (10)	45.6	8.5	14.2	3.6	1.9	0.6	8.0	2.1	9.9	5.1	14.9	7.9	8.1	1.4	18.8	2.0
9 (3)	51.2	11.8	17.3	2.9	1.8	0.8	10.3	2.6	9.7	2.5	15.0	7.0	7.0	0.9	16.0	5.3
12 (7)	60.5	7.7	19.3	3.6	2.2	0.4	9.2	2.6	7.9	3.3	19.4	5.3	9.1	1.9	15.9	2.0
13 (10)	62.4	11.0	18.6	5.1	2.1	0.7	9.4	3.1	12.7	3.7	28.2	11.5	7.8	1.1	16.1	3.7
16 (3)	59.5	3.0	15.3	1.5	2.5	0.3	6.1	0.8	11.3	6.0	32.7	16.3	10.2	0.8	14.3	1.5
17 (4)	64.4	6.4	16.6	4.6	3.3	1.5	5.6	1.6	3.8	2.2	13.5	5.4	8.5	0.8	16.0	3.5
18 (10)	68.5	11.4	15.7	2.7	2.3	0.7	7.2	1.4	7.6	3.8	16.5	7.5	9.4	1.0	20.9	2.3
19 (10)	56.0	8.6	17.5	2.6	2.1	0.4	8.6	1.4	9.1	5.5	20.2	13.5	8.2	1.4	17.7	2.1
20 (9)	50.6	11.3	13.0	2.3	2.0	0.5	7.1	2.9	6.1	3.3	9.4	6.1	8.2	1.1	18.7	2.6
21 (10)	48.1	5.3	14.3	1.9	1.8	0.4	8.2	1.5	8.2	4.3	11.6	4.4	8.7	1.8	19.0	4.2
22 (4)	48.4	7.8	17.1	0.3	1.6	0.5	11.6	3.8	10.3	5.0	12.5	6.2	8.1	2.2	21.0	2.4
24 (6)	51.7	10.2	14.7	4.5	1.7	0.4	9.1	3.7	10.5	5.4	13.5	7.3	9.0	2.2	16.8	3.7
25 (10)	65.2	10.2	16.0	2.9	1.4	0.3	11.8	3.1	11.8	7.1	27.0	15.6	8.9	1.8	16.6	3.1
26 (9)	57.1	8.3	11.9	2.7	1.9	0.7	6.7	1.6	6.2	3.6	10.9	7.3	9.3	1.6	19.8	2.9
27 (7)	43.1	11.4	11.5	4.7	1.6	0.7	7.4	2.0	8.6	5.0	14.9	13.0	7.7	2.1	17.1	4.5
33 (10)	49.8	5.3	14.9	4.4	2.0	0.4	7.8	3.0	6.7	4.0	8.9	3.6	9.6	2.0	21.3	4.4
34 (10)	39.0	7.4	12.3	1.1	1.7	0.4	7.3	1.5	5.2	2.5	6.3	3.7	8.8	1.8	18.0	3.8
37 (9)	27.8	9.0	9.1	1.2	1.4	0.3	7.1	1.3	1.8	1.6	1.8	1.6	8.3	2.7	16.9	3.7
38 (7)	39.1	14.3	10.8	3.5	1.8	0.5	6.3	1.0	3.4	2.4	6.0	4.5	8.7	2.4	20.3	3.5

39 (9)	34.2	10.3	10.3	1.8	1.7	0.5	6.7	2.9	2.3	1.9	3.3	3.1	7.8	1.1	15.8	2.4
40 (6)	42.3	14.2	11.7	4.1	1.6	0.6	7.5	0.8	3.7	3.9	5.8	5.8	8.2	1.7	17.8	2.0
41 (9)	48.4	8.6	14.2	3.2	2.4	1.2	6.6	1.7	3.4	2.6	7.2	6.2	10.0	1.6	14.7	1.5
42 (7)	50.6	4.0	15.5	4.2	2.2	0.3	7.1	1.2	9.4	2.4	15.9	6.0	10.3	1.1	18.9	2.4
43 (3)	29.2	1.5	13.9	1.0	2.8	0.7	5.2	1.2	2.7	1.5	3.7	1.5	8.8	1.6	20.0	1.0
44 (10)	39.8	15.8	13.4	4.1	2.0	0.6	6.9	1.6	5.6	3.0	8.3	5.9	10.5	2.1	19.7	2.1
45 (10)	64.3	7.5	12.2	1.7	1.9	0.4	6.8	2.1	5.6	4.3	10.3	6.2	10.4	1.3	20.2	2.1
46 (10)	45.5	6.9	13.4	3.3	1.8	0.5	7.5	1.7	4.5	2.9	7.2	3.7	8.8	1.2	17.5	3.5
47 (10)	45.7	6.7	14.2	2.4	2.1	0.2	6.9	1.5	2.6	2.7	4.2	3.1	9.4	1.2	16.7	3.4
48 (9)	40.6	5.7	15.0	3.7	2.0	0.4	7.6	1.3	3.1	1.4	3.8	1.5	9.6	1.6	17.3	2.2
52 (9)	57.6	7.2	17.1	3.9	2.7	0.7	6.6	1.9	2.0	1.1	4.9	2.4	10.4	1.3	17.8	4.5
59 (3)	50.3	4.9	13.0	4.5	2.3	1.0	6.2	3.4	1.7	1.2	2.3	0.6	8.5	1.5	16.0	3.6
60 (7)	51.6	3.6	12.9	4.3	2.4	0.7	5.6	1.9	1.4	0.8	2.3	0.8	9.6	1.9	18.4	1.6
63 (6)	52.6	5.7	12.2	2.8	2.2	0.7	5.7	1.4	1.3	0.8	2.7	0.8	9.0	1.5	22.2	3.4
64 (3)	50.5	6.6	14.3	2.0	2.7	0.3	5.4	1.2	1.0	0.0	3.0	1.7	8.5	1.3	18.3	1.5
65 (10)	53.3	10.6	15.0	3.0	2.7	0.9	6.2	2.6	2.8	2.1	4.8	3.0	9.5	1.3	20.2	2.7
Average	51.1	8.5	14.3	3.1	2.1	0.6	7.4	2	6.2	3.5	11.5	6.5	8.9	1.6	18.1	3.1

Table S4: Pearson's correlation matrix between cynarin, echinacoside and Dodeca-2E,4E,8Z,10E/Z-tetraenoic isobutylamide % quantitation results (%) and morpho-quantitative variables.

Values in bold type differ from 0 at a significance level α=0.05

	Plant height (cm)	Leaf length (cm)	Leaf width (cm)	L/W leaf ratio of basal rosette	N° stems /plant	N° flowers/ plant	Ø main flower (cm)	N° ray flowers/ main flower head	Root weight (g)	Cynarin (%)	Echina coside (%)	Dodeca (%)	Absolute Cynarin (mg)	Absolute Echinaco side (mg)	Absolute Dodeca (mg)	N° plant on 15/10/13	N° plants with flower stems on 15/10/13	N° plants with flowers on 15/10/13	N° plants on (with or without flowers) 05/08/14
Plant height (cm)	1	**0.59**	.301	.236	**0.462**	**0.675**	.218	-.026	**0.502**	.211	**-0.414**	**-0.329**	**0.547**	**0.353**	.182	**0.476**	**0.569**	**0.46**	.216
leaf length (cm)	**0.59**	1	**0.343**	**0.507**	**0.494**	**0.573**	.090	-.181	**0.332**	.110	-.238	**-0.4**	**0.331**	.279	.024	.251	**0.526**	.379*	.070
Leaf width (cm)	.301	**0.343**	1	**-0.593**	-.301	-.043	**-0.19**	-.036	.116	**0.511**	-.171	-.077	**0.375**	.024	.074	.131	.267	.148	.102
L/W leaf ratio of basal rosette	.236	**0.507**	**-0.593**	1	**0.667**	**0.504**	**0.217**	-.117	.163	**-0.356**	-.025	-.299	-.073	.205	-.092	.099	.256	.280	-.051
N° stem/plant	**0.462**	**0.494**	-.301	**0.667**	1	**0.874**	**0.314**	-.116	**0.416**	-.108	**-0.333**	**-0.35**	.290	.257	.120	.217	**0.507**	**0.495**	-.034
N° flowers/plant	**0.675**	**0.573**	-.043	**0.504**	**0.874**	1	**0.352**	-.274	**0.434**	.178	**-0.382**	**-0.463**	**0.491**	.259	.006	**0.347**	**0.729**	**0.619**	-.014
Ø main flower (cm)	.218	.090	**-0.19**	**0.217**	**0.314**	**0.352**	1	-.081	.288	-.191	-.208	.042	.193	.219	.254	**0.764**	**0.327**	**0.34**	**0.5**
N° ray flowers/main flower head	-.026	-.181	-.036	-.117	-.116	-.274	-.081	1	-.005	-.274	.242	.222	-.193	.135	.229	.004	-.283	-.262	.115
Root weight (g)	**0.502**	**0.332**	.116	.163	**0.416**	**0.434**	.288	-.005	1	.077	**-0.351**	-.178	**0.824**	**0.847**	**0.708**	**0.323**	.230	.155	.263
Cynarin (%)	.211	.110	**0.511**	**-0.356**	-.108	.178	-.191	-.274	.077	1	-.039	-.092	**0.598**	.031	-.049	-.007	.306	.143	-.227

Echinacoside (%)	**-0.414**	-.238	-.171	-.025	**-0.333**	**-0.382**	-.208	.242	**-0.351**	-.039	1	.298	**-0.316**	.164	-.082	**-0.322**	-.276	-.233	-.270
Dodeca (%)	**-0.329**	**-0.4**	-.077	-.299	**-0.35**	**-0.463**	.042	.222	-.178	-.092	.298	1	-.223	-.031	**0.528**	-.065	**-0.424**	**-0.381**	.130
Absolute Cynarin (mg)	**0.547**	**0.331**	**0.375**	-.073	.290	**0.491**	.193	-.193	**0.824**	**0.598**	**-0.316**	-.223	1	**0.672**	**0.504**	.299	**0.417**	.248	.064
Absolute Echinacoside (mg)	**0.353**	.279	.024	.205	.257	.259	.219	.135	**0.847**	.031	.164	-.031	**0.672**	1	**0.678**	.181	.096	.028	.136
Absolute Dodeca (mg)	.182	.024	.074	-.092	.120	.006	.254	.229	**0.708**	-.049	-.082	**0.528**	**0.504**	**0.678**	1	.230	-.146	-.175	**0.351**
N° plant on 15/10/13	**0.476**	.251	.131	.099	.217	**0.347**	**0.764**	.004	**0.323**	-.007	**-0.322**	-.065	.299	.181	.230	1	**0.451**	**0.412**	**0.627**
N° plants with flower stems on 15/10/13	**0.569**	**0.526**	.267	.256	**0.507**	**0.729**	**0.327**	-.283	.230	.306	-.276	**-0.424**	**0.417**	.096	-.146	**0.451**	1	**0.858**	-.073
N° plants with flowers on 15/10/13	**0.46**	**0.379**	.148	.280	**0.495**	**0.619**	**0.34**	-.262	.155	.143	-.233	**-0.381**	.248	.028	-.175	**0.412**	**0.858**	1	-.036
N° plants on (with or without flowers) 05/08/14	.216	.070	.102	-.051	-.034	-.014	**0.5**	.115	.263	-.227	-.270	.130	.064	.136	**0.351**	**0.627**	-.073	-.036	1
